# Design, synthesis, and biological evaluation of a novel series of 2-(2,6-dioxopiperidin-3-yl)isoquinoline-1,3(2*H*,4*H*)-dione derivatives as cereblon modulators

**DOI:** 10.1080/14756366.2022.2087219

**Published:** 2022-06-14

**Authors:** Yilin Liu, Yuming Song, Yingju Xu, Meixu Jiang, Haibin Lu

**Affiliations:** a College of Pharmacy, Jilin University, Changchun, China; b Department of VIP Unit, China-Japan Union Hospital, Jilin University, Changchun, China

**Keywords:** Anticancer, CRBN, IMiDs, NCI-H929

## Abstract

In the current study, we designed and synthesised a novel series of 2-(2,6-dioxopiperidin-3-yl)isoquinoline-1,3(2*H*,4*H*)-dione derivatives as cereblon (CRBN) modulators. The results of the CCK8 assay revealed potent antiproliferative activity for the selected compound **10a** against NCI-H929 (IC_50_=2.25 µM) and U239 (IC_50_=5.86 µM) cell lines. Compound **10a** also can inhibit the TNF-α level (IC_50_=0.76 µM) in LPS stimulated PMBC and showed nearly no toxicity to this normal human cell line. The TR-FRET assay showed compound **10a** having potent inhibitory activity against CRBN (IC_50_=4.83 µM), and the docking study confirmed a nice fitting of **10a** into the active sites of CRBN. Further biology studies revealed compound **10a** can increase the apoptotic events, arrest the NCI-H929 cells at G0/G1 cell cycle, and induce the ubiquitination degradation of IKZF1 and IKZF3 proteins by CRL4^CRBN^. These preliminary results suggested that compound **10a** could serve as a potential antitumor drug and worthy of further investigation.

## 1. Introduction

Multiple myeloma (MM) is a malignant blood neoplasm characterised by an abnormal intramedullary proliferation of bone marrow cells and hypersecretion of monoclonal immunoglobulins^1^^,^^2^. It accounts for 10% of all haematologic malignancies and generally occurs between 40 and 70 years of life^3–5^. The immunomodulatory drugs (IMiDs), such as lenalidomide, a new class of anticancer agents with the glutarimide group are clinically effective in the treatment of MM^6–8^. These drugs can inhibit the production of many inflammatory mediators such as tumour necrosis factor-alpha (TNF-α), IL-1, IL-2, IL-4, IL-5, IL-6, IL-10, and interferon-γ (IFN-γ), inhibiting the secretion of beta fibroblast growth factor (bFGF) and vascular endothelial growth factor (VEGF)^9^^,^^10^, showing pleiotropic effects on MM cells and their microenvironment, promoting cell apoptosis, interfering with the production of cell adhesion factors, regulating the production of cytokines and inhibiting the production of tumour related angiogenesis^11–13^.

**Figure 1.**
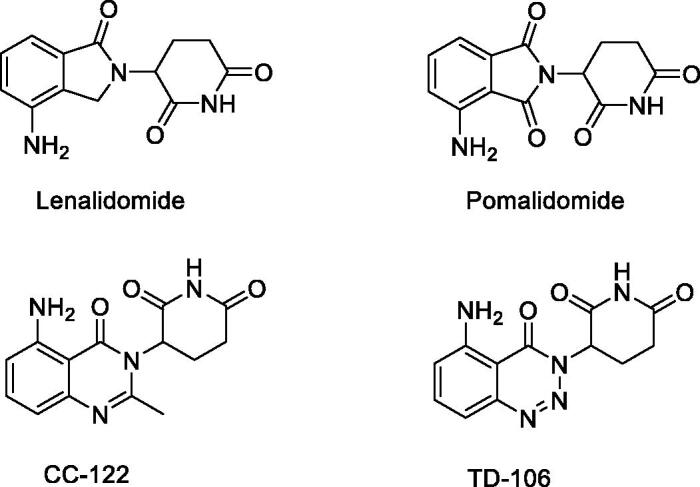
Chemical structures of CRBN modulators.

Cereblon (CRBN), the molecular target of these IMiDs, is a substrate receptor for the CRL4 (CUL4–RBX1–DDB1) ubiquitin ligase complex^14–17^. CRBN ligand binding confers neomorphic activity, altering the substrate specificity of the ubiquitin ligase by promoting the recruitment of substrate proteins^18–21^. Once binding to CRBN, IMiDs promote the degradation of IKZF1 and IKZF3 through the ubiquitination dependent proteasome pathway, thereby driving the clinical activity in MM^22–26^. Thalidomide is the first IMiD approved for the treatment of MM. As thalidomide functioned successfully as an IMiD^27^^,^^28^, next generation IMiDs, such as lenalidomide^29^^,^^30^, pomalidomide^31^^,^^32^, CC-122^33^^,^^34^, and TD-106^35^, which have a good effect on MM, were developed (Figure 1).

**Figure 2.**
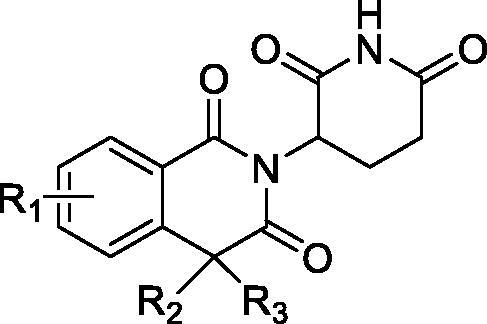
Chemical structures of design CRBN modulators.

The crystal structure of CRBN-DDB1 binding to lenalidomide shows mechanistic insight into how IMiDs act on CRL4^CRBN^. The IMiD compounds bind CRBN through their shared glutarimide ring, leaving portions of their variable phthaloyl ring solvent-exposed^14^. In this study, we describe the discovery of a series of isoquinoline-1,3(2*H*,4*H*)-dione derivatives as a type of novel CRBN modulator, which retain the glutarimide group and enlarge the five membered ring in the middle of the compound to six membered ring (Figure 2). The SAR of all the newly synthesised compounds were studied by the proliferation assay. The TNF-α inhibition ability and toxicity to normal human cells were also investigated. The most potent compound **10a** was selected to be further studied through the TR-FRET assay and molecular docking to identify its CRBN binding activity. Furthermore, the effect of **10a** on the induction of apoptosis and cell cycle on NCI-H929 cell line were investigated using flow cytometry. The IKZF1 and IKZF3 proteins degradation ability of **10a** was also investigated by immunoblot.

## 2. Results and discussion

### 2.1. Chemistry

**Scheme 1.**
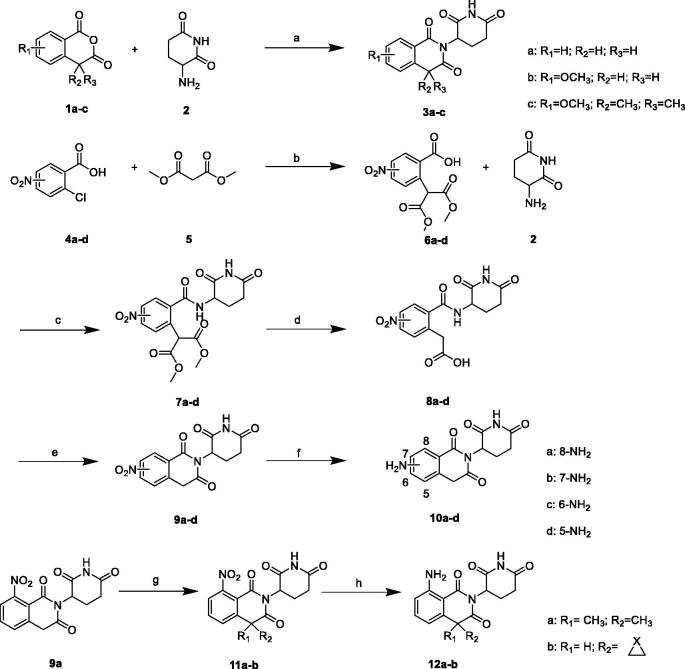
Reagents and conditions: (a) CH_3_COONa, CH_3_COOH, reflux, 24 h; (b) CH_3_ONa, CuBr, 80 °C, 24 h; (c) TBTU, DIPEA, DCM, rt, overnight; (d) DMSO, 10% NaOH, rt, 6 h; (e) CH_3_COOH, reflux, 12 h; (f) SnCl_2_·2H_2_O, CH_3_OH; (g) K_2_CO_3_, DMF, alkyl halide, rt, 4 h; (h) SnCl_2_·2H_2_O, CH_3_OH.

The synthetic route for 2-(2,6-dioxopiperidin-3-yl)isoquinoline-1,3(2*H*,4*H*)-dione derivatives is depicted in Scheme 1. Briefly, compounds **3a–c** were synthesised from the commercial homophthalic anhydride derivatives and reacted with 3-aminopiperidine-2,6-dione hydrochloride under acetic acid. The compounds **9a** and **10a–d** were prepared from the nitro substituted 2-chlorobenzoic acid **4a–d**. The compounds **4a–d** first reacted with dimethyl malonate under the CuBr to obtain the compounds **6a–d**. Compounds **6a–d** amidated with 3-aminopiperidine-2,6-dione hydrochloride, and then decarboxylation under NaOH and cyclised under acetic acid condition to get the compounds **9a–d**. The nitro group of the compounds **9a–d** were reduced by stannous chloride to obtain the target compounds **10a–d**. Compounds **12a, b** were prepared from the reaction of compound **9a** with alkyl halides and then reduced the nitro group.

### 2.2. Biological evaluation

#### 2.2.1. Antiproliferative activity

All the new compounds were evaluated for their antiproliferative activities against NCI-H929 and U2932 cancer cell lines and contrasted with lenalidomide using CCK8 assay. The results revealed the ability of the new compounds to inhibit the growth of the selected cancer cell lines with IC_50_ values (Table 1).

**Table 1.** Antiproliferative activity and TNF-α inhibition in LPS stimulated human PBMC of the compounds.

Comp. no	IC_50_ (μM ± SD)^a^			PBMC cell viability (%)^b^
	NCI-H929	U2932	TNF-α	
**3a**	32.24 ± 1.42	>50	>50	99
**3b**	28.22 ± 0.92	36.38 ± 1.22	43.84 ± 1.88	98
**3c**	>50	>50	>50	100
**9a**	9.26 ± 0.56	12.24 ± 0.58	5.48 ± 1.04	94
**10a**	2.25 ± 0.09	5.86 ± 0.12	0.76 ± 0.08	99
**10b**	16.28 ± 0.56	20.56 ± 0.82	21.28 ± 1.26	98
**10c**	18.65 ± 0.83	26.32 ± 0.76	38.46 ± 1.38	100
**10d**	>50	>50	>50	99
**12a**	>50	>50	>50	100
**12b**	>50	>50	>50	100
Lenalidomide	1.12 ± 0.06	3.24 ± 0.11	0.13 ± 0.02	86

^a^IC_50_: the half maximal inhibitory concentration.

^b^Cell viability measured by the CCK-8. The viable cell number was expressed as a percentage relative to control cells.

Among these derivatives, 8-amino substituted compound **10a** was the most active against NCI-H929 cells (2.25 ± 0.09 µM) and U2932 cells (5.86 ± 0.12 µM), showed comparable activity with lenalidomide against NCI-H929 cells (1.12 ± 0.06 µM) and U2932 cells (3.24 ± 0.11 µM). Changing the amino group to 6 (compound **10b**) or 7 (compound **10c**) substitution position the antiproliferative activities weakened, and the 5-amino substituted compound **10d** was the weakest (IC_50_>50 µM). On the other hand, the antiproliferative activities decreased significantly (IC_50_>50 µM) if the four positions of the derivatives were substituted by alkyl group (compounds **12a, b and 3c**).

Replacing the amino group to other groups, such as compounds **3a** (hydrogen), **3c** (7-methoxy), and **9a** (8-nitro) substituted, the activities also decreased, compared with compound **10a**. Therefore, we chose compound **10a** for further biological activity study and the molecular docking investigation.

#### 2.2.2. PMBC toxicity and TNF-α inhibition assay

Previous studies have reported IMiDs stimulate immunomodulatory activity main through the TNF-α inhibition^9^^,^^10^. In this test, the effect of the synthesised compounds on TNF-α in LPS stimulated human peripheral blood mononuclear cell (PBMC) was evaluated (Table 1). The SAR of TNF-α inhibition was similar to the antiproliferative activity. Compound **10a** showed remarkable significant reduction in TNF-α level at IC_50_=0.76 ± 0.08 µM, compared with lenalidomide IC_50_=0.13 ± 0.02 µM.

And same time, the toxicity of the compounds on the normal human cell line PBMC was also tested by CCK8. All the compounds showed nearly no toxicity at the 20.0 µM concentration. The PMBC cell viability of the most active compound **10a** (94%) was better than lenalidomide (86%) at the same concentration.

#### 2.2.3. TR-FRET analysis

**Figure 3.**
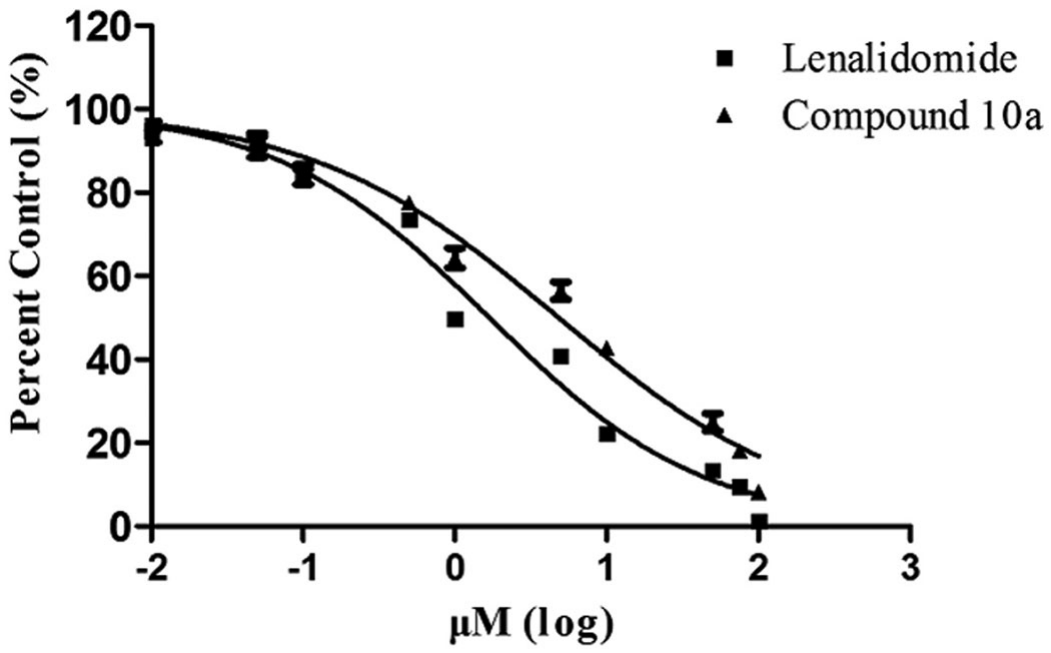
Determination of the relative cereblon binding affinities for compound **10a** and lenalidomide by TR-FRET.

To determine the relative binding affinities between lenalidomide and compound **10a**, we used a TR-FRET CRBN binding assay to determine the IC_50_ values for these compounds. The assay monitors the displacement of the Cy5-labeled thalidomide from the tri-trp pocket of CRBN. Under these assay conditions, the IC_50_ value for compound **10a** was at 4.83 µM, compared with lenalidomide IC_50_=1.69 µM (Figure 3). Proved compound **10a** having high binding affinity to CRBN protein.

#### 2.2.4. Annexin V-FITC/PI apoptosis assay

**Figure 4.**
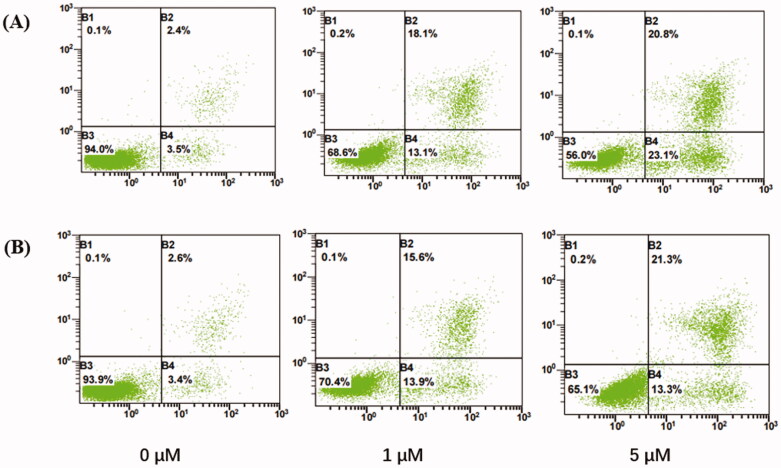
Annexin V phases of NCI-H929 treated with (A) lenalidomide and (B) compounds **10a** at the indicated concentrations (72 h, *x*-axis: Annexin V; *y*-axis: PI). B1: necrotic cells; B2: late apoptosis; B3: live cells; B4: early apoptosis.

The IMiDs were reported to induce apoptosis through activation of caspase 8 in MM cells^36^. Accordingly, the ability of the selected compound **10a** to induce apoptosis in NCI-H929 cells was investigated using annexin V fluorescein isothiocyanate (FITC)/propidium iodide (PI) staining assay. NCI-H929 cells were treated with compound **10a** and lenalidomide for 72 h. The results are presented in Figure 4.

The results revealed a significant dose dependent increase of the apoptotic events by compound **10a** from 6.0% in the control, to 29.5% and 34.6% at 0, 1, and 5 µM concentration, and be equivalent to lenalidomide from 5.9% in the control, to 31.2% and 43.9%. We can see the significant increase in the percentages of early apoptosis from 3.4% to 13.3% and late apoptosis from 2.6% to 21.3%. Confirmed compound **10a** can induce NCI-H929 cells apoptosis.

#### 2.2.5. Cell cycle analysis

**Figure 5.**
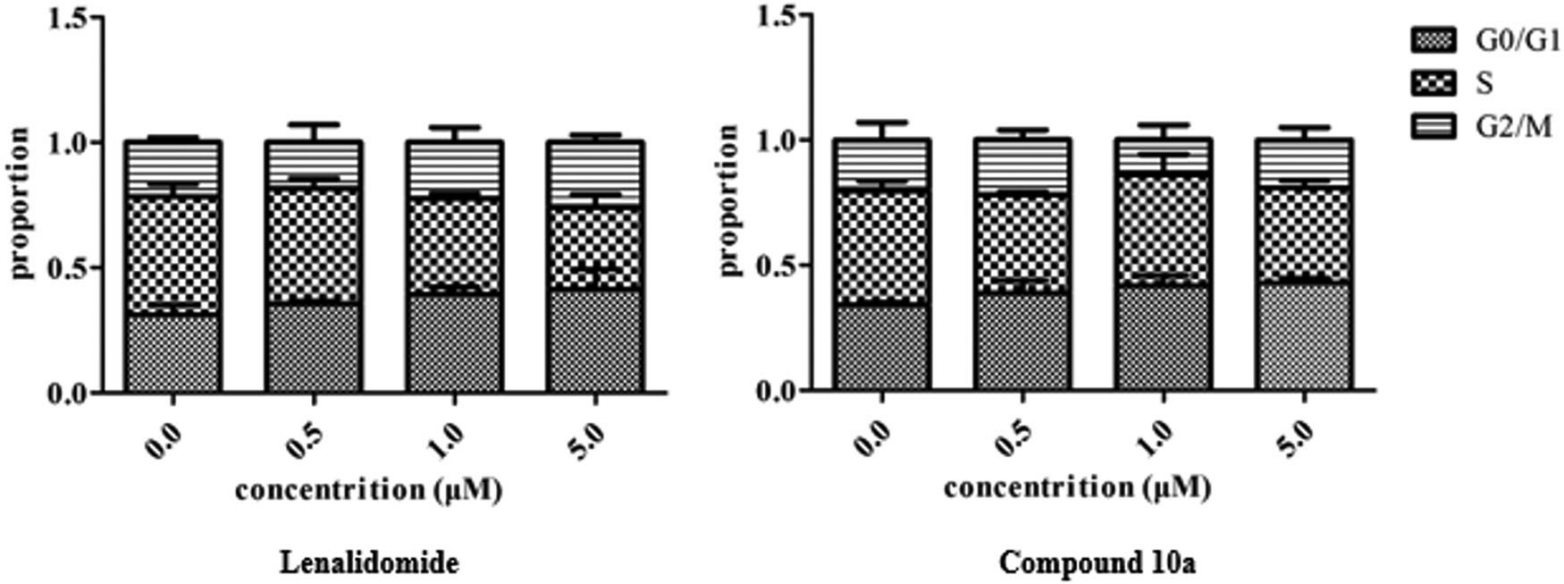
Flow cytometry bar chart showing the effect of compound **10a** and lenalidomide at the indicated concentrations on cell cycle distribution of NCI-H929 cells after treatment for 48 h.

To better understand the mechanism by which compound **10a** inhibits MM cell growth, cell cycle analysis was performed with the selected compound **10a** and contrasted with lenalidomide. The NCI-H929 cells were treated with each of the compounds at 0, 0.5, 1.0, and 5.0 µM for 48 h. Following this treatment, the PI-stained cells were analysed and the results are outlined in Figure 5.

The results of the cell cycle analysis revealed that compound **10a** and lenalidomide can induce G0/G1 cell cycle arrest. The cell cycle arrest ability of **10a** showed a dose-dependent manner in the G0/G1 cell cycle from 34.0% to 42.9% at 0 and 5.0 µM concentrations.

#### 2.2.6. Immunoblot analysis

**Figure 6.**
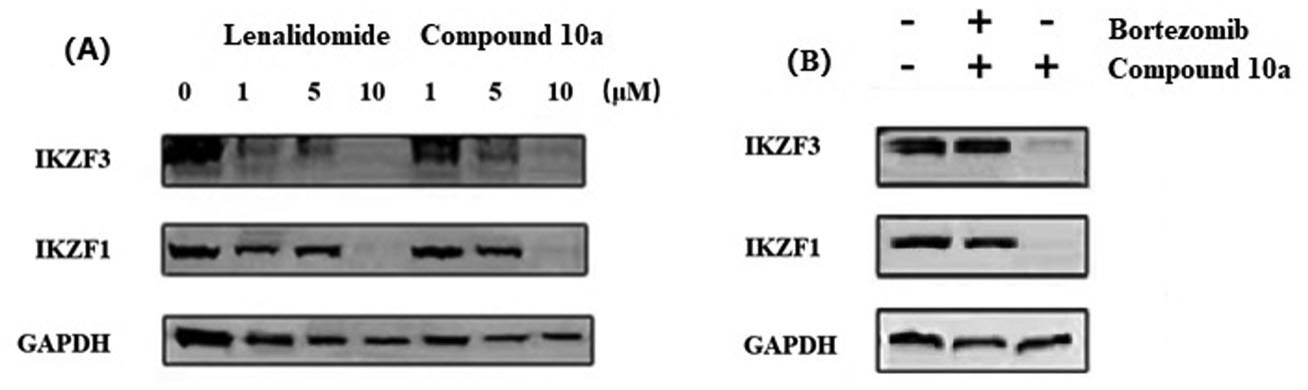
Immunoblot analysis of (A) whole cell extracts of NCI-H929 cells incubated for 72 h with lenalidomide or compound **10a** at the indicated concentrations; (B) NCI-H929 cells treated with compound **10a** (10 μM) alone or in combination with bortezomib (100 nm) for 72 h.

The earlier studies have revealed that the CRBN modulators thalidomide, lenalidomide, and pomalidomide can induce the ubiquitination of IKZF1 and IKZF3 by CRL4^CRBN^. Subsequent proteasome degradation of these transcription factors kills MM cells. Accordingly, we used the immunoblot assay to measure the IKZF1 and IKZF3 proteins degradation in the current study. The results revealed that compound **10a** induced the degradation of IKZF1 and IKZF3 as lenalidomide (Figure 6(A)). The treatment with compound **10a** resulted in the loss of IKZF1 and IKZF3 levels with a significant dose dependent, and can completely degrade IKZF1 and IKZF3 at 10 µM concentration for 72 h in NCI-H929 cells.

To further explore this phenomenon, we treated the NCI-H929 cells with compound **10a** (10 µM) alone or in combination with the proteasome inhibitor bortezomib (100 nm)^37^^,^^38^. The Western blot results (Figure 6(B)) showed that beginning 1 h at 100 nM bortezomib treatment can block the degradation of IKZF1/3, confirmed the proteins degradation was mediated by the proteasome.

### 2.3. Docking study into CRBN

**Figure 7.**
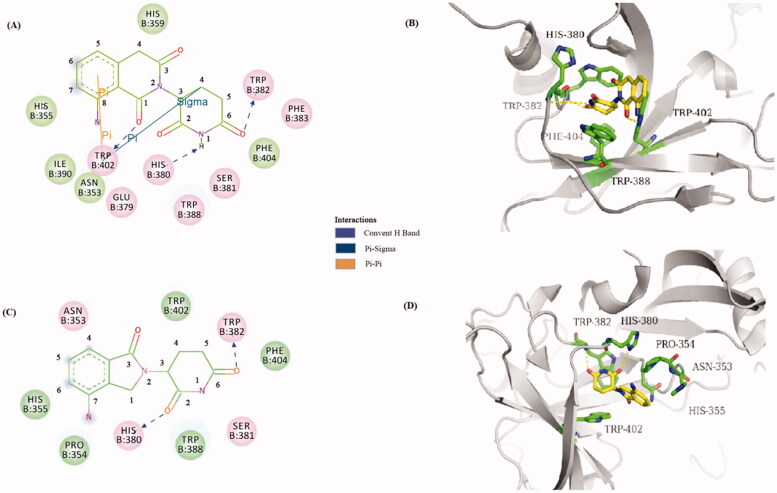
Binding modes and interactions of compound **10a** and lenalidomide into CRBN (pdb: 4CI2): (A) 2D model of the interaction between compound **10a** and the amino acid residues of CRBN protein. (B) 3D model of the binding position of compound **10a**. (C) 2D model of the interaction between lenalidomide and the amino acid residues of CRBN protein. (D) 3D model of the binding position of compound lenalidomide.

To elucidate whether compound **10a** targeted the CRBN protein, we carried out a molecular docking study to predict the possible binding mode of compound **10a** with the CRBN. The outcomes of the molecular docking study showed that the binding mode of compound **10a** within the binding pocket of CRBN (binding energy of –7.5 kcal/mol). The glutarimide group is held in a buried cavity between CRBN sheets β10 and β13, which has the similar binding pose with lenalidomide as the literature report (Figure 7)^14^. Furthermore, the 8-aminoisoquinoline-1,3(2*H*,4*H*)-dione carbonyl (C1), the glutarimide carbonyl (C6) and the intervening amide (N1) are in hydrogen-bonding distance to CRBN residues Trp402, Trp382, and His380, respectively. By comparison to lenalidomide, the glutarimide carbonyls (C2 and C6) are in hydrogen-bonding distance to CRBN residues His380 and Trp382, respectively. The molecular docking analyses indicated that compound **10a** binds snugly into the active sites of CRBN.

## 3. Conclusions

In summary, we designed and synthesised a novel series of 2-(2,6-dioxopiperidin-3-yl)isoquinoline-1,3(2*H*,4*H*)-dione derivatives as new kinds of CRBN modulators. Study on the SAR of the derivatives based on cell proliferation assay, which resulted in the discovery of compound **10a** with considerably antiproliferative potency against MM cell lines NCI-H929 (IC_50_=2.25 µM) and U239 (IC_50_=5.86 µM), and nearly no toxicity to the normal human cell line PMBC at 20 µM concentration. Compound **10a** also can reduce the TNF-α level (IC_50_=0.76 µM) in LPS stimulated PMBC. The TR-FRET analysis and molecular docking study results of compound **10a** agreed with its ability of inhibiting CRBN and predicted binding mode. Further biology studies revealed compound **10a** can increase the apoptotic events, arrest the NCI-H929 cells at G0/G1 cell cycle, and induce the degradation of IKZF1 and IKZF3 proteins degradation by CRL4^CRBN^. Our findings suggested that compound **10a** could be considered as a potential anti-MM drug candidate or as a novel CRBN modulator which can be used for targeted protein degradation for further development.

## 4. Experimental

### 4.1. Chemistry

#### 4.1.1. Materials and methods

Unless otherwise noted, all reagents and solvents were obtained from commercially available sources and were used without purification. ^1^H NMR spectra were tested in CDCl_3_ or DMSO-d_6_ with TMS as the internal reference on a Bruker AVANCE 400 (Billerica, MA). Mass spectra (MS) were obtained from Agilent 1100 mass spectrometer (Santa Clara, CA) with an electron spray ionisation source.

#### 4.1.2. Synthesis of 2-(2,6-dioxopiperidin-3-yl)isoquinoline-1,3(2H,4H)-dione (3a)

A mixture of homophthalic anhydride **1a** (1.0 g, 6.2 mmol), 3-aminopiperidine-2,6-dione hydrochloride **2** (1.0 g, 6.1 mmol), and sodium acetate anhydrous (0.85 g, 6.2 mmol) was added in acetic acid 50 mL. The resulting mixture was heated to reflux for 24 h. After cooling to room temperature, acetic acid was evaporated and the residue was purified by flash column chromatography (methanol:dichloromethane = 1:20) to obtain compound **3a** as a slightly yellow solid (1.5 g, 90%). ^1^H NMR (400 MHz, DMSO-d_6_): 10.94 (s, 1H), 8.12–7.98 (m, 1H), 7.70 (t, *J* = 7.4 Hz, 1H), 7.53–7.47 (m, 1H), 7.42 (d, *J* = 7.6 Hz, 1H), 5.66–5.58 (m, 1H), 4.37–4.13 (m, 2H), 2.92–2.83 (m, 1H), 2.57–2.43 (m, 2H), and 1.96–1.90 (m, 1H); LCMS [M + H]^+^: 273.03.

#### 4.1.3. 2-(2,6-Dioxopiperidin-3-yl)-7-methoxyisoquinoline-1,3(2H,4H)-dione (3b)

It was prepared as for **3a** as a yellow solid, 85% yield. ^1^H NMR (400 MHz, DMSO-d_6_): 10.91 (s, 1H), 7.51 (d, *J* = 39.2 Hz, 1H), 7.36–7.31 (m, 2H), 5.65–5.55 (m, 1H), 4.27–3.99 (m, 2H), 3.82 (s, 3H), 2.92–2.83 (m, 1H), 2.56–2.40 (m, 2H), and 1.99–1.91 (m, 1H); LCMS [M + H]^+^: 303.03.

#### 4.1.4. 2-(2,6-Dioxopiperidin-3-yl)-7-methoxy-4,4-dimethylisoquinoline-1,3(2H,4H)-dione (3c)

It was prepared as for **3a** as a yellow solid, 85% yield. ^1^H NMR (400 MHz, DMSO-d_6_): 10.93 (d, *J* = 13.0 Hz, 1H), 7.66 (d, *J* = 8.0 Hz, 1H), 7.57–7.48 (m, 1H), 7.36–7.33 (m, 1H), 5.65–5.54 (m, 1H), 3.85 (s, 3H), 2.93–2.83 (m, 1H), 2.57–2.35 (m, 2H), 1.97–1.94 (m, 1H), and 1.62–1.49 (m, 6H); LCMS [M + H]^+^: 331.06.

#### 4.1.5. Synthesis of 2-(1,3-dimethoxy-1,3-dioxopropan-2-yl)-6-nitrobenzoic acid (6a)

To a solution of dimethyl malonate, 15 mL was added 2-chloro-6-nitro-benzoic acid **4a** (1.0 g, 5.0 mmol) and sodium methanolate (0.8 g, 15.0 mmol) under nitrogen. The mixture was stirred at room temperature for 30 min, then added cuprous bromide (0.12 g, 0.8 mmol). The resulting mixture was heated at 80 °C for 24 h. After cooling to room temperature, water 50 mL was added to the mixture followed by hexanes 50 mL. The aqueous layer was separated and then added toluene 50 mL, and the biphasic mixture was filtered through celite to remove insolubles. Then the aqueous layer was separated and acidified with 6 N aqueous HCl to pH 2–3, and then extracted twice with ethyl acetate. The combined organic phase was washed with brine and dried over anhydrous Na_2_SO_4_. After filtration and evaporation, the crude residue was purified by flash column chromatography (ethyl acetate:petroleum ether = 1:2) to obtain **6a** (0.9 g, 61%) as a yellow oil. ^1^H NMR (400 MHz, CDCl_3_): 8.15 (d, *J* = 8.2 Hz, 1H), 7.99 (d, *J* = 7.9 Hz, 1H), 7.68 (t, *J* = 8.1 Hz, 1H), 5.07 (s, 1H), and 3.81 (s, 6H).

#### 4.1.6. 2-(1,3-Dimethoxy-1,3-dioxopropan-2-yl)-5-nitrobenzoic acid (6b)

It was prepared as for **6a** as a yellow solid, 54% yield. ^1^H NMR (400 MHz, DMSO-d_6_): 8.67 (d, *J* = 2.6 Hz, 1H), 8.45–8.42 (m, 1H), 7.67 (d, *J* = 8.5 Hz, 1H), 5.88 (s, 1H), and 3.72 (s, 6H).

#### 4.1.7. 2-(1,3-Dimethoxy-1,3-dioxopropan-2-yl)-4-nitrobenzoic acid (6c)

It was prepared as for **6a** as a yellow solid, 68% yield. ^1^H NMR (400 MHz, CDCl_3_): 8.36 (s, 1H), 8.29 (s, 2H), 5.82 (s, 1H), and 3.81 (s, 6H).

#### 4.1.8. 2-(1,3-Dimethoxy-1,3-dioxopropan-2-yl)-3-nitrobenzoic acid (6d)

It was prepared as for **6a** as a yellow solid, 60% yield. ^1^H NMR (400 MHz, DMSO-d_6_): 8.26–8.23 (m, 2H), 7.74 (t, *J* = 8.0 Hz, 1H), 6.07 (s, 1H), and 3.63 (s, 6H).

#### 4.1.9. Synthesis of dimethyl 2-(2-((2,6-dioxopiperidin-3-yl)carbamoyl)-3-nitrophenyl)malonate (8a)

Compound **6a** (1.0 g, 2.5 mmol), TBTU (1.6 g, 5.0 mmol), DIPEA (1.4 g, 10.8 mmol), and 3-aminopiperidine-2,6-dione hydrochloride (0.7 g, 4.0 mmol) were added into dichloromethane 50 mL, the mixture reacted under room temperature overnight. Then water 50 mL was added to the solution and the aqueous layer was extracted twice with dichloromethane. The combined organic phase was combined and dried to give the crude compound **7a**, which was used for the next reaction without further purification.

The compound **7a** was dissolved in DMSO 5 mL, and then added into 10% NaOH aqueous solution 1 mL. The mixture reacted under room temperature for 6 h, and then water 20 mL was added and acidified with concentrated HCl to pH 2. The white precipitate was filtered and washed twice with methanol 10 mL to obtain compound **8a** (0.5 g, 44%) as a white solid. ^1^H NMR (400 MHz, DMSO-d_6_): 10.86 (s, 1H), 8.46 (s, 1H), 7.59 (s, 1H), 7.19 (s, 1H), 5.71 (s, 1H), 3.82–3.72 (m, 3H), 2.96–2.78 (m, 1H), 2.56–2.55 (m, 1H), and 1.90 (s, 1H).

#### 4.1.10. 2-(2-((2,6-Dioxopiperidin-3-yl)carbamoyl)-4-nitrophenyl)acetic acid (8b)

It was prepared as for **8a** as a yellow solid, 53% yield. ^1^H NMR (400 MHz, DMSO-d_6_): 10.94 (d, *J* = 14.0 Hz, 1H), 8.70 (d, *J* = 42.8 Hz, 1H), 8.19 (s, 2H), 5.92–5.73 (m, 1H), 3.85 (d, *J* = 19.5 Hz, 3H), 2.94–2.84 (m, 1H), 2.60–2.50 (m, 1H), and 2.02–1.92 (m, 1H).

#### 4.1.11. 2-(2-((2,6-Dioxopiperidin-3-yl)carbamoyl)-5-methylphenyl)acetic acid (8c)

It was prepared as for **8a** as a yellow solid, 44% yield. ^1^H NMR (400 MHz, DMSO-d_6_): 11.02 (s, 1H), 9.21 (s, 1H), 8.31–8.24 (m, 1H), 7.96 (s, 1H), 6.01–5.72 (m, 1H), 4.05–3.84 (m, 1H), 2.98–2.85 (m, 1H), 2.62–2.50 (m, 2H), and 2.04–1.99 (m, 1H).

#### 4.1.12. 2-(2-((2,6-Dioxopiperidin-3-yl)carbamoyl)-6-nitrophenyl)acetic acid (8d)

It was prepared as for **8a** as a yellow solid, 40% yield. ^1^H NMR (400 MHz, DMSO-d_6_): 11.00 (s, 1H), 8.48-8.43 (m, 2H), 7.89 (s, 1H), 5.64–5.53 (m, 1H), 3.68 (s, 2H), 2.91–2.82 (m, 1H), 2.58–2.39 (m, 2H), and 1.99–1.91 (m, 1H).

#### 4.1.13. Synthesis of 2-(2,6-dioxopiperidin-3-yl)-8-nitroisoquinoline-1,3(2H,4H)-dione (9a)

Compound **8a** (1.0 g, 3.0 mmol) was added into acetic acid 30 mL and stirred at reflux for 12 h. After cooling to room temperature, acetic acid was evaporated and the residue was purified by flash column chromatography (methanol:dichloromethane = 1:20) to obtain compound **9a** (0.8 g, 85%) as a slightly yellow solid. ^1^H NMR (400 MHz, DMSO-d_6_): 10.95 (s, 1H), 7.89 (t, *J* = 7.8 Hz, 1H), 7.78 (t, *J* = 9.9 Hz, 1H), 7.66 (m, 1H), 5.58 (s, 1H), 4.50–4.32 (m, 2H), 3.37 (s, 1H), 2.88–2.79 (m, 1H), 2.57–2.49 (m, 1H), 2.41–2.31 (m, 1H), and 1.95–1.88 (m, 1H); LCMS [M + H]^+^: 318.01.

#### 4.1.14. 2-(2,6-Dioxopiperidin-3-yl)-7-nitroisoquinoline-1,3(2H,4H)-dione (9b)

It was prepared as for **9a** as a yellow solid, 82% yield. ^1^H NMR (400 MHz, DMSO-d_6_): 11.00 (s, 1H), 8.70 (d, *J* = 40.0 Hz, 1H), 8.50 (d, *J* = 7.7 Hz, 1H), 7.72 (s, 1H), 5.68–5.62 (m, 1H), 4.51–4.39 (m, 2H), 2.94–2.85 (m, 1H), 2.60–2.47 (m, 2H), and 1.99–1.92 (m, 1H).

#### 4.1.15. 2-(2,6-Dioxopiperidin-3-yl)-6-nitroisoquinoline-1,3(2H,4H)-dione (9c)

It was prepared as for **9a** as a yellow solid, 80% yield. ^1^H NMR (400 MHz, DMSO-d_6_): 10.98 (m, 1H), 8.33–8.21 (m, 3H), 5.65–5.60 (m, 1H), 4.48–4.36 (m, 2H), 2.93–2.84 (m, 1H), 2.59–2.44 (m, 2H), and 1.98–1.91 (m, 1H).

#### 4.1.16. 2-(2,6-Dioxopiperidin-3-yl)-5-nitroisoquinoline-1,3(2H,4H)-dione (9d)

It was prepared as for **9a** as a yellow solid, 74% yield. ^1^H NMR (400 MHz, DMSO-d_6_): 10.98 (s, 1H), 8.54–8.41 (m, 2H), 7.80–7.76 (m, 1H), 5.65–5.61 (m, 1H), 4.67–4.46 (m, 2H), 2.93–2.84 (m, 1H), 2.58–2.49 (m, 2H), and 1.99–1.92 (m, 1H).

#### 4.1.17. Synthesis of 8-amino-2-(2,6-dioxopiperidin-3-yl)isoquinoline-1,3(2H,4H)-dione (10a)

Compound **9a** (100 mg, 0.32 mmol) and stannous chloride dihydrate (360 mg, 1.6 mmol) were added into methanol 5 mL and stirred at reflux for 6 h. The solution was evaporated and added into water 20 mL, and alkalised by ammonium hydroxide to pH = 7. The aqueous solution was extracted by dichloromethane (3 × 20 mL), then the combined organic phase was washed with brine and dried over anhydrous Na_2_SO_4_. After filtration and evaporation, the crude residue was purified by flash column chromatography (methanol:dichloromethane = 1:20) to obtain **10a** (27 mg, 33%) as a yellow solid. ^1^H NMR (400 MHz, DMSO-d_6_): 10.89 (s, 1H), 7.28–7.22 (m, 1H), 7.10 (s, 1H), 6.69 (dd, *J* = 16.0, 8.4 Hz, 1H), 6.41 (dd, *J* = 7.2, 0.9 Hz, 1H), 5.61–5.52 (m, 1H), 4.12–3.92 (m, 2H), 2.89–2.82 (m, 1H), 2.56–2.37 (m, 2H), and 1.97–1.87 (m, 1H); LCMS [M + H]^+^: 288.08.

#### 4.1.18. 7-Amino-2-(2,6-dioxopiperidin-3-yl)isoquinoline-1,3(2H,4H)-dione (10b)

It was prepared as for **10a** as a yellow solid, 30% yield. ^1^H NMR (400 MHz, DMSO-d_6_): 10.91 (d, *J* = 8.0 Hz, 1H), 7.32–7.18 (m, 1H), 7.06 (d, *J* = 11.2 Hz, 1H), 6.91 (d, *J* = 8.0 Hz, 1H), 5.63–5.55 (m, 1H), 4.07–3.89 (m, 2H), 2.91–2.82 (m, 1H), 2.57–2.38 (m, 2H), and 1.94–1.87 (m, 1H); LCMS [M + H]^+^: 288.08.

#### 4.1.19. 6-Amino-2–(2,6-dioxopiperidin-3-yl)isoquinoline-1,3(2H,4H)-dione (10c)

It was prepared as for **10a** as a yellow solid, 33% yield. ^1^H NMR (400 MHz, DMSO-d_6_): 10.85 (s, 1H), 7.89–7.62 (m, 1H), 6.80–6.23 (m, 2H), 5.59–5.50 (m, 1H), 4.18–3.96 (m, 2H), 2.87–2.81 (m, 1H), 2.54–2.43 (m, 2H), and 1.87 (brs, 1H); LCMS [M + H]^+^: 288.08.

#### 4.1.20. 5-Amino-2–(2,6-dioxopiperidin-3-yl)isoquinoline-1,3(2H,4H)-dione (10d)

It was prepared as for **10a** as a yellow solid, 28% yield. ^1^H NMR (400 MHz, DMSO-d_6_): 10.91 (s, 1H), 7.38–7.16 (m, 2H), 6.96–6.94 (m, 1H), 5.63–5.56 (m, 1H), 3.88–3.76 (m, 2H), 2.91–2.81 (m, 1H), 2.57–2.39 (m, 2H), and 1.94–1.88 (m, 1H); LCMS [M + H]^+^: 288.08.

#### 4.1.21. 8-Amino-2–(2,6-dioxopiperidin-3-yl)-4,4-dimethylisoquinoline-1,3(2H,4H)-dione (12a)

It was prepared as for **10a** as a yellow solid, 34% yield. ^1^H NMR (400 MHz, DMSO-d_6_): 10.89 (s, 1H), 7.34–7.29 (m, 1H), 6.77–6.70 (m, 2H), 5.75–5.59 (m, 1H), 2.97–2.88 (m, 1H), 2.74–2.67 (m, 1H), 2.47–2.32 (m, 1H), 1.95–1.90 (m, 1H), and 1.52 (dd, *J* = 31.1, 7.2 Hz, 6H); LCMS [M + H]^+^: 316.06.

#### 4.1.22. 8′-Amino-2′-(2,6-dioxopiperidin-3-yl)-1′H-spiro[cyclopropane-1,4′-isoquinoline]-1′,3′(2′H)-dione (12b)

It was prepared as for **10a** as a yellow solid, 41% yield. ^1^H NMR (400 MHz, DMSO-d_6_): 10.89 (d, *J* = 16.0 Hz, 1H), 7.27–7.22 (m, 1H), 6.69–6.63 (m, 2H), 6.11–6.08 (m, 1H), 5.69–5.56 (m, 1H), 2.90–2.81 (m, 1H), 2.55–2.53 (m, 1H), 2.46–2.32 (m, 1H), and 1.96–1.61 (m, 5H); LCMS [M + H]^+^: 314.04.

#### 4.1.23. Synthesis of 2-(2,6-dioxopiperidin-3-yl)-4,4-dimethyl-8-nitroisoquinoline-1,3(2H,4H)-dione (11a)

Compound **9a** (1.0 g, 3.2 mmol), methyl iodide (0.91 g, 6.4 mmol), and potassium carbonate (0.88 g, 6.4 mmol) were added into DMF 10 mL. The mixture was stirred at room temperature for 4 h, then water 20 mL was added and extracted by dichloromethane (3 × 20 mL). The organic phase was washed with brine and dried over anhydrous Na_2_SO_4_. After filtration and evaporation, the crude residue was purified by flash column chromatography **(**methanol:dichloromethane = 1:20**)** to obtain **11a** (0.7 g, 63%) as a yellow solid. ^1^H NMR (400 MHz, DMSO-d_6_): 10.97 (s, 1H), 8.03 (d, *J* = 4.0 Hz, 1H), 7.96 (t, *J* = 7.9 Hz, 1H), 7.84 (d, *J* = 4.0 Hz, 1H), 5.55 (dd, *J* = 11.7, 5.1 Hz, 1H), 2.89–2.80 (m, 1H), 2.43–2.30 (m, 1H), 2.01–1.88 (m, 1H), and 1.70–1.56 (m, 6H).

##### 4.1.24. 2′-(2,6-Dioxopiperidin-3-yl)-8′-nitro-1′H-spiro[cyclopropane-1,4′-isoquinoline]-1′,3′(2′H)-dione (11b)

It was prepared as for **11a** as a yellow solid, 56% yield. ^1^H NMR (400 MHz, DMSO-d_6_): 10.96 (d, *J* = 8.0 Hz, 1H), 7.80–7.73 (m, 1H), 7.38 (d, *J* = 8.0 Hz, 1H), 5.65–5.58 (m, 1H), 2.89–2.79 (m, 1H), 2.56–2.53 (m, 1H), 2.44–2.33 (m, 2H), 2.11–2.10 (m, 1H), and 1.98–1.93 (m, 4H).

### 4.2. Biological evaluation

The NCI-H929 and U2932 cells were purchased from American Type Culture Collection (ATCC) (Manassas, VA). Lenalidomide and bortezomib were purchased from Macklin Biochemical Co., Ltd. (Shanghai, China). IKZF1 antibody (#9034) and IKZF3 antibody (#15103) were obtained from Cell Signaling Technology (Danvers, MA). Cell counting kit-8 (CCK-8), Annexin V-FITC apoptosis detection kit, and BCA protein assay kit, DAPI were purchased from Beyotime (Nantong, China). IRDye^®^680 goat anti-mouse IgG was purchased from Li-COR Biosciences Inc. (Lincoln, NE).

#### 4.2.1. Cell proliferation, apoptosis, and cell cycle assay^39^

The NCI-H929 cells were cultured in RPMI-1640 media supplemented with 10% FBS, 100 U/mL penicillin–100 µg/mL streptomycin, and 0.05 mM 2-mercaptoethanol. The U2932 cells were cultured in RPMI-1640 media supplemented with 10% FBS and 100 U/mL penicillin–100 µg/mL streptomycin. The PBMCs were isolated from healthy donors.

For the cell proliferation experiments, 1.0 × 10^5^ cells were first seeded in the 96-well culture plate and incubated overnight. Compounds tested were serially diluted in the corresponding media and then added to the well of 96-well plate in a final volume of 100 µL and then the cells were incubated for 72 h at 37 °C. Finally, 10 µL of CCK-8 was added to each well for 1–4 h, and the absorbance values were read at 450 nm in a Microplate Reader (ELx-800, BioTek Instruments, Winooski, VT). The IC_50_ value for each compound was calculated at the basis of the number of viable cells using GraphPad Prism 5.0 software (San Diego, CA).

Flow cytometry was used to analyse effects of the compounds tested on cell cycle (PI staining) and apoptosis (Annexin V-FITC and PI staining). Stained cells were analysed on a Flow Cytometer (BD Biosciences, Franklin Lakes, NJ), and the data were analysed using the Cell Quest software.

#### 4.2.2. TNF-α ELISA assay^40^

PBMC (4 × 10^5^ cells) were incubated in the 96-well culture plate and stimulated by 2 µg/mL LPS. The level of TNF-α was checked by standard ELISA (R&D Systems, Minneapolis, MN). Analysis was performed by following the manufacture’s procedure for each ELISA kit. The test samples were assessed in triplicate and absorbance was taken on a Microplate Reader (ELx-800, BioTek Instruments, Winooski, VT) at 450 nm. The IC_50_ value for each compound was calculated by comparison with standard curves with purified recombinant TNF-α using GraphPad Prism 5.0 software (San Diego, CA).

#### 4.2.3. Immunoblot analysis

For immunoblot analysis, NCI-H929 cells were treated with the tested compounds at the indicated concentrations for various times, collected, and lysed in RIPA buffer in the presence of protease inhibitors on ice for 30 min. The protein from each sample was quantitated using a BCA protein assay kit, and was separated by SDS-PAGE and then transferred onto the PVDF membrane. The membranes were probed with specific primary antibodies at 4 °C overnight, followed by incubation with IRDye^®^680 goat anti-mouse secondary antibodies. The signals were acquired using the Odyssey Infrared Imaging System (LI-COR, Lincoln, NE).

#### 4.2.4. TR-FRET assay^41^^,^^42^

His-tagged CRBN-DDB1 complex (Abcam, Cambridge, UK, catalogue no. ab235611) 60 nM was mixed with Eu-anti-His Tag antibody 3 nM (Thermo Fisher, Waltham, MA, catalogue no. PV5596) in a final buffer containing 20 mM HEPES pH 7.0, 150 mM NaCl, and 0.005% Tween-20. The solution was then mixed with Cy5-labeled thalidomide 10 nM and various concentrations of compounds, and then was incubated at room temperature for 1 h. FRET signals were measured on an EnVision plate reader by exciting at 340 nm and recording emission at both 615 nm (no FRET control) and 665 nm (FRET signals) with a 60 µs delay. FRET efficiency was calculated by the ratio of 665 nm/615 nm. Quantitative loss of FRET efficiency as a function of compound concentrations was fitted by GraphPad Prism 5.0 (San Diego, CA) and calculated the IC_50_.

### 4.3. Molecular docking studies

The docking studies were done using AutoDock 4.2.6. During the docking simulations, the pdb file of CRBN (pdb: 4CI2) was downloaded from protein data bank (http://www.rcsb.org/pdb) and the ligand and its single bonds were moved freely within the potential binding pocket. Discovery studio and PyMOL softwares were used to visualise the binding interaction. The result of the docking study of the compound **10a** is represented in Figure 7.

## Supplementary Material

Supplemental Material
