# Supplementary material for: Design, synthesis, and biological evaluation of a novel series of 2-(2,6-dioxopiperidin-3-yl)isoquinoline-1,3(2H,4H)-dione derivatives as cereblon modulators
Source: J Enzyme Inhib Med Chem. 2022 Jun 14;37(1):1715–23. doi: 10.1080/14756366.2022.2087219 (PMC9225785; doi:10.1080/14756366.2022.2087219)
Supplement: Supplemental Material [file IENZ_A_2087219_SM9654.pdf]

Supporting information

Design, synthesis, and biological evaluation of a novel series of 2-(2,6-dioxopiperidin-3-yl)isoquinoline-1,3(2*H*,4*H*)-dione derivatives as cereblon modulators

Yilin Liu[a,*], Yuming Song[b,*], Yingju Xu[a], Meixu Jiang[a], Haibin Lu[a]

[a] College of Pharmacy, Jilin University, Changchun,China;

[b] Department of VIP Unit, China-Japan Union Hospital, Jilin University, Changchun, China.

Content

NMR and MS spectra of the final compounds……………………………….2-11

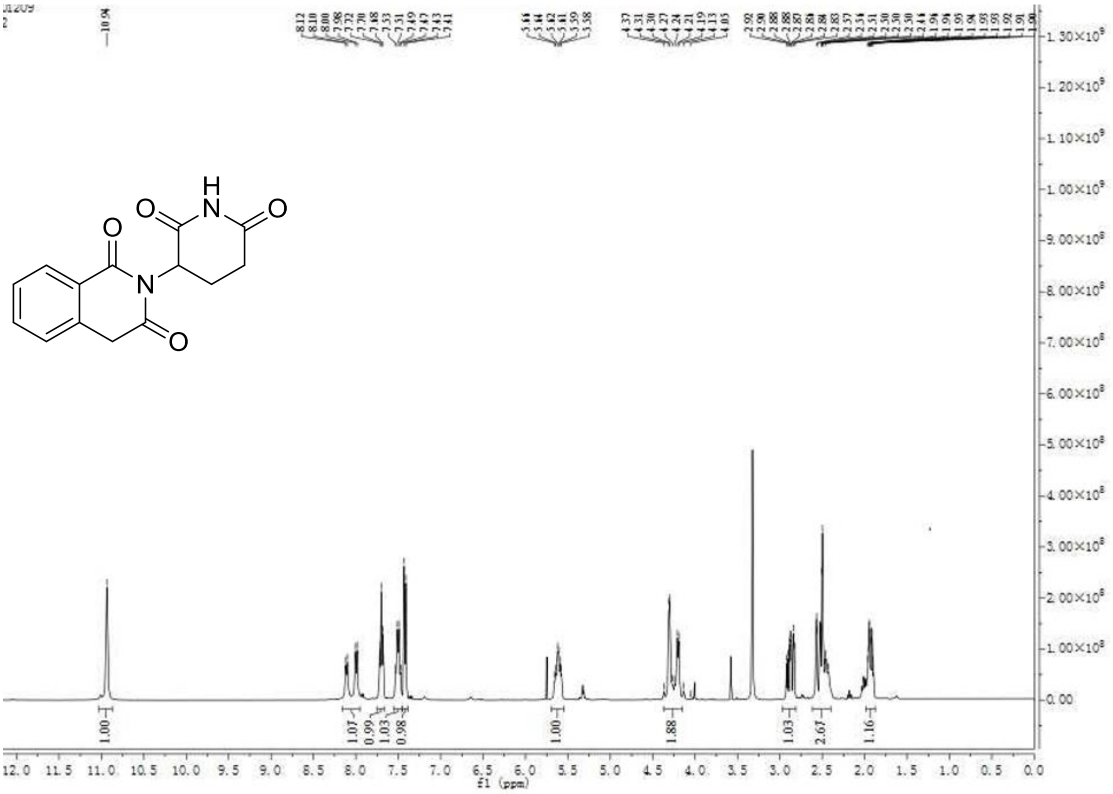

Figure S1. ^{1}H NMR spectrum of compound 3a

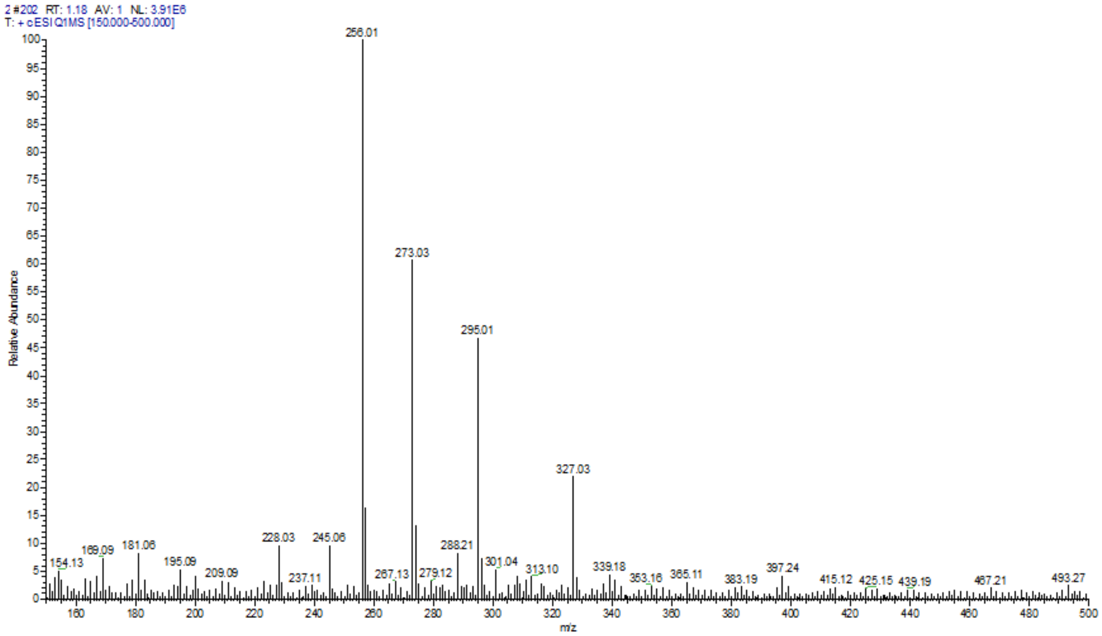

Figure S2. MS spectrum of compound 3a

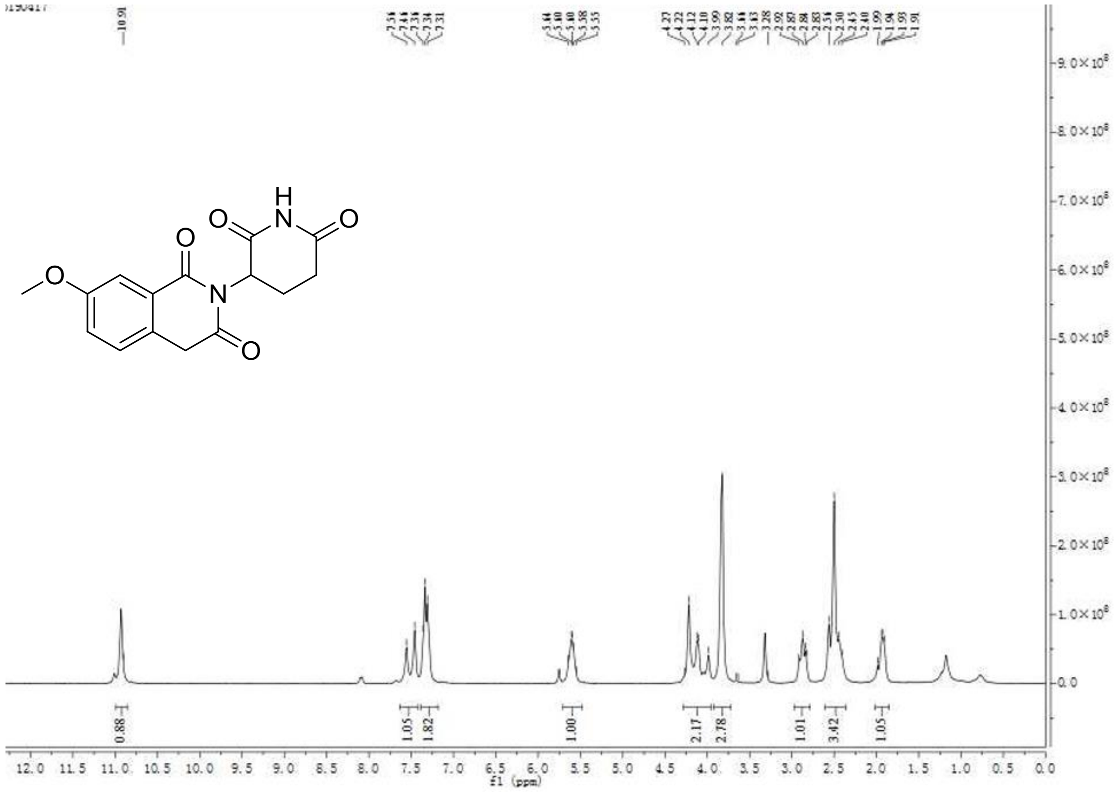

Figure S3. ^{1}H NMR spectrum of compound 3b

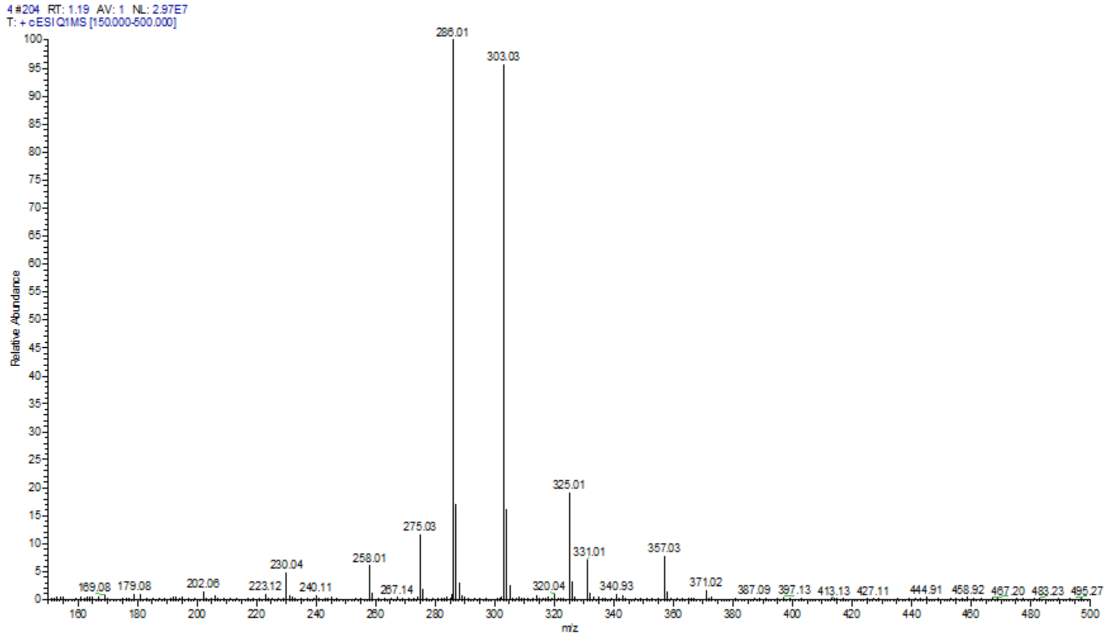

Figure S4. MS spectrum of compound 3b

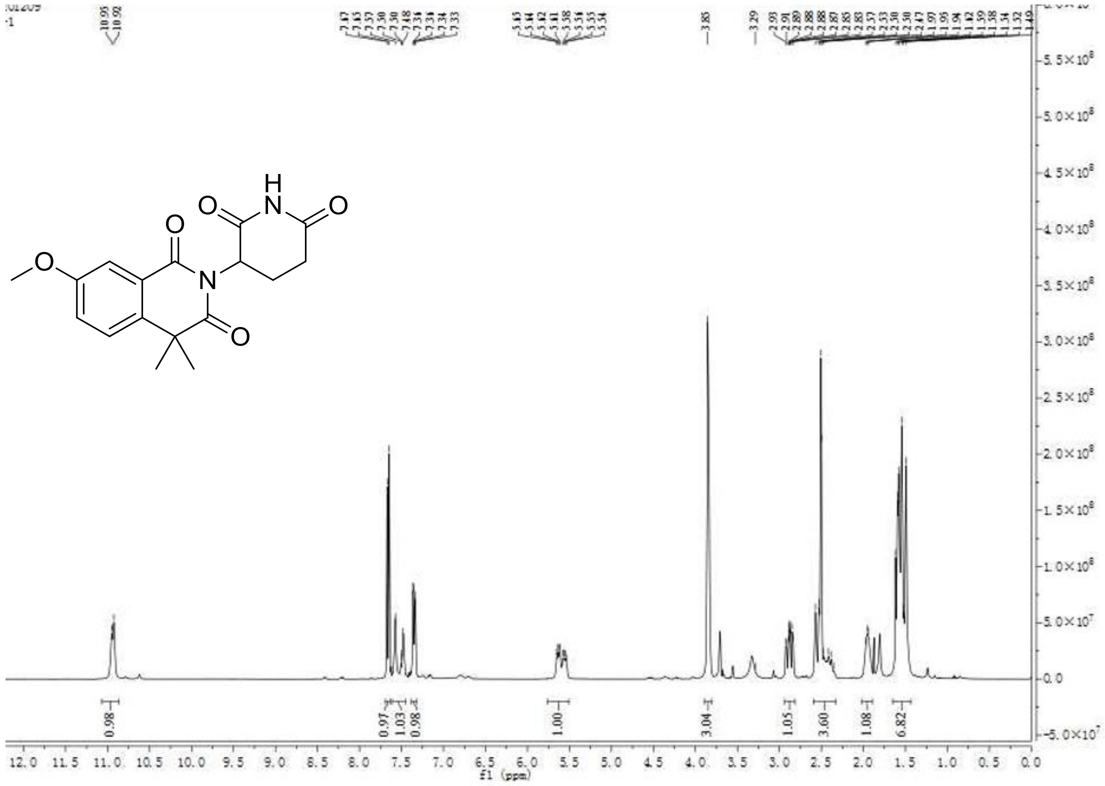

Figure S5. ^{1}H NMR spectrum of compound 3c

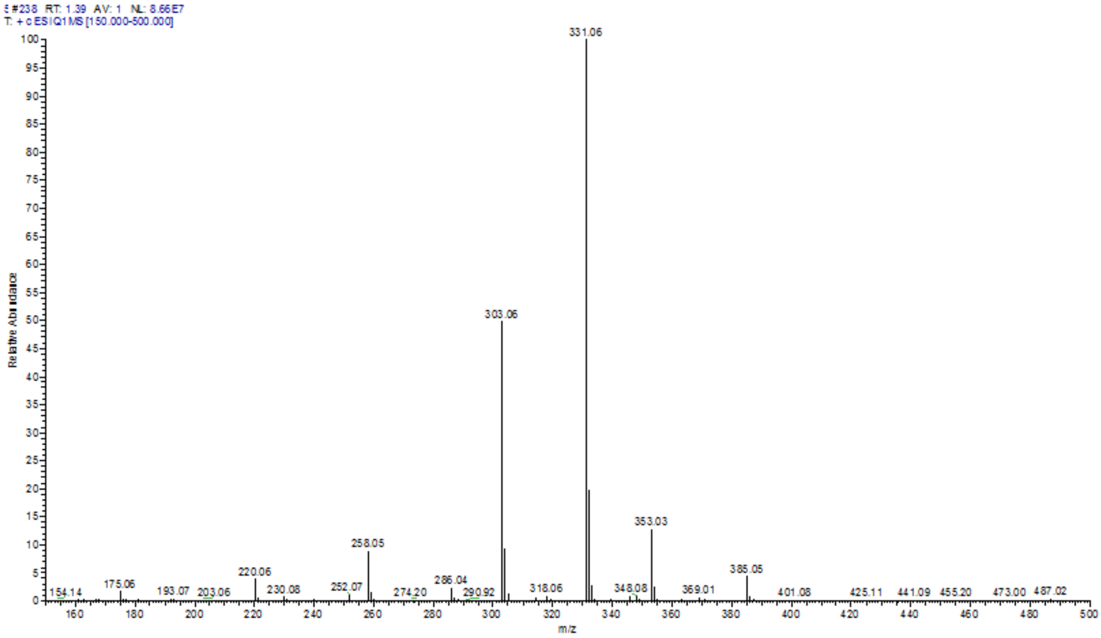

Figure S6. MS spectrum of compound 3c

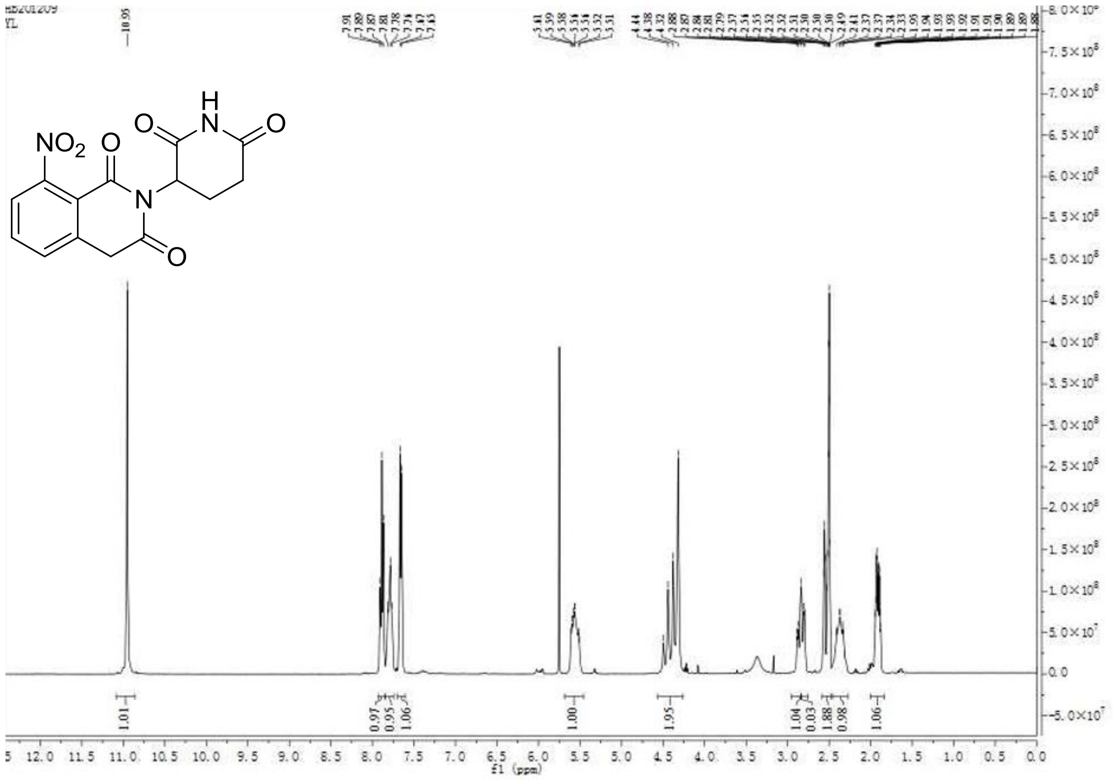

Figure S7. ^{1}H NMR spectrum of compound 9a

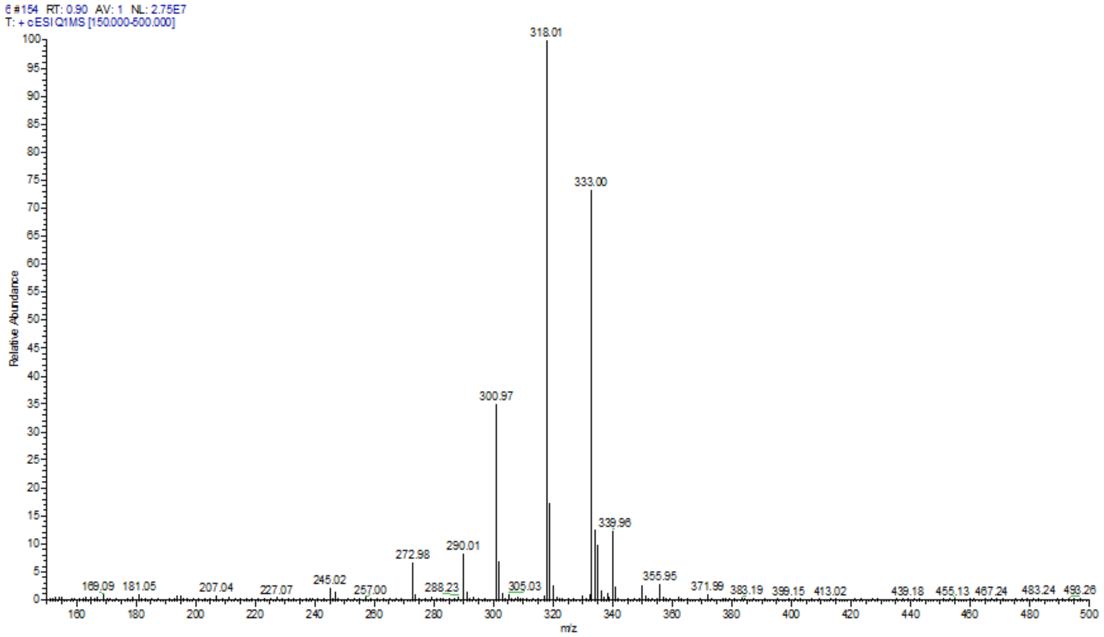

Figure S8. MS spectrum of compound 9a

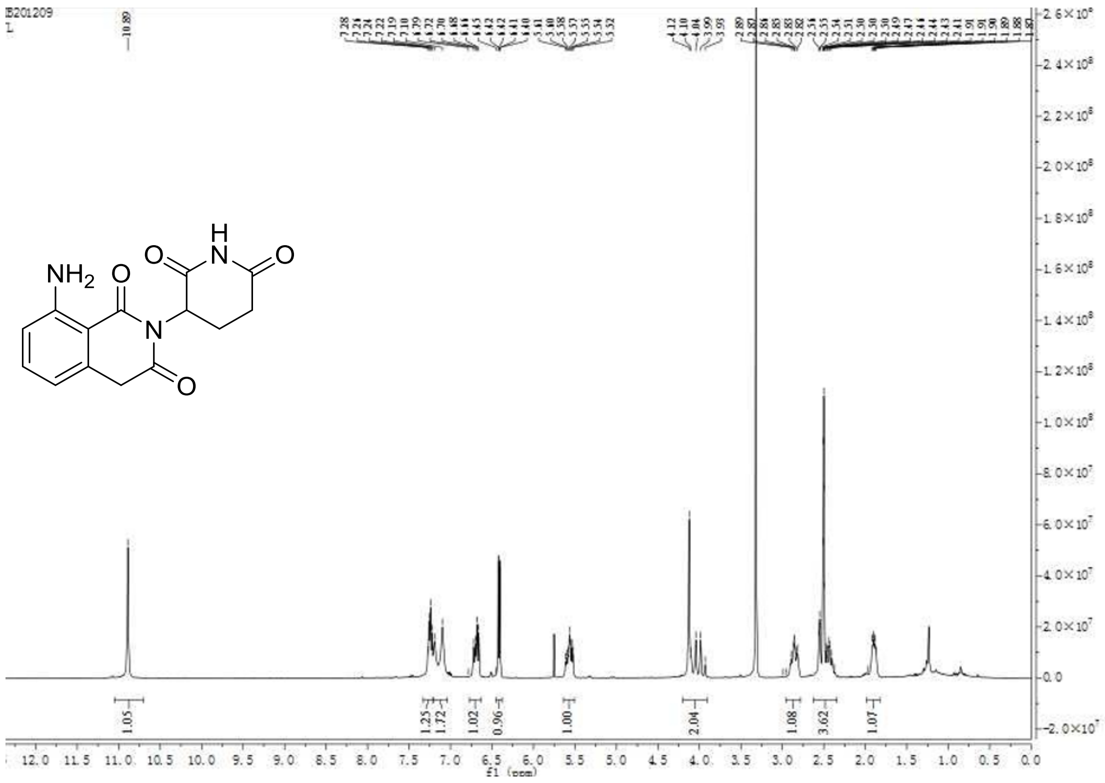

Figure S9. ^{1}H NMR spectrum of compound 10a

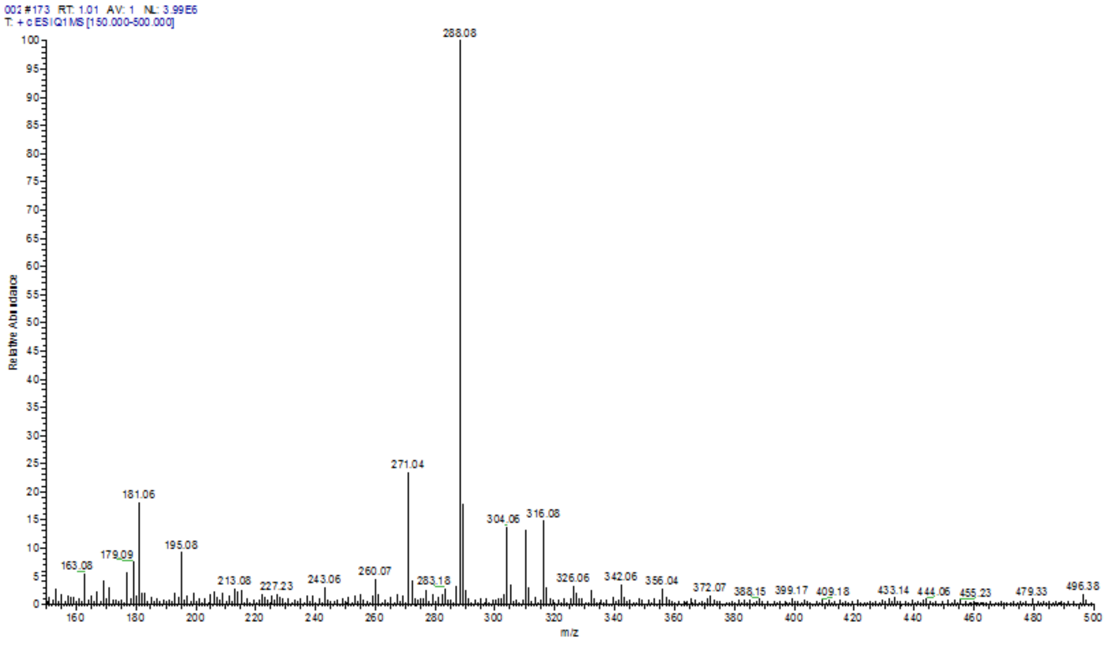

Figure S10. MS spectrum of compound 10a

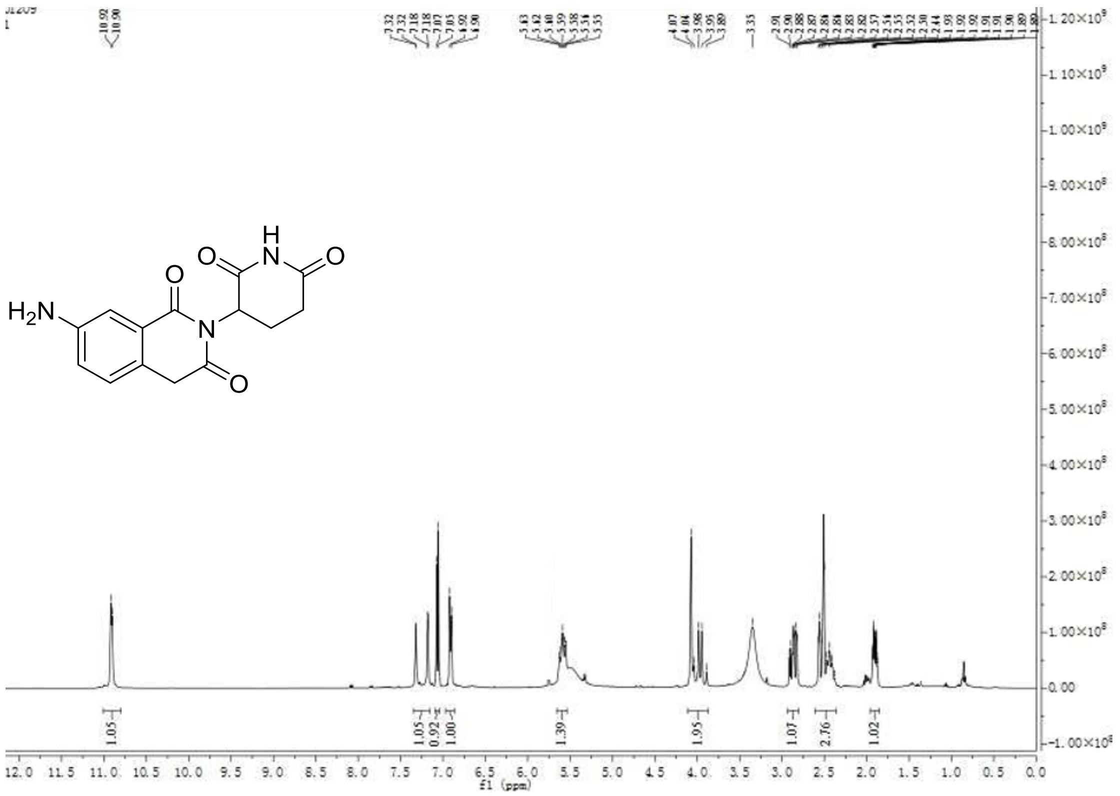

Figure S11. ^{1}H NMR spectrum of compound 10b

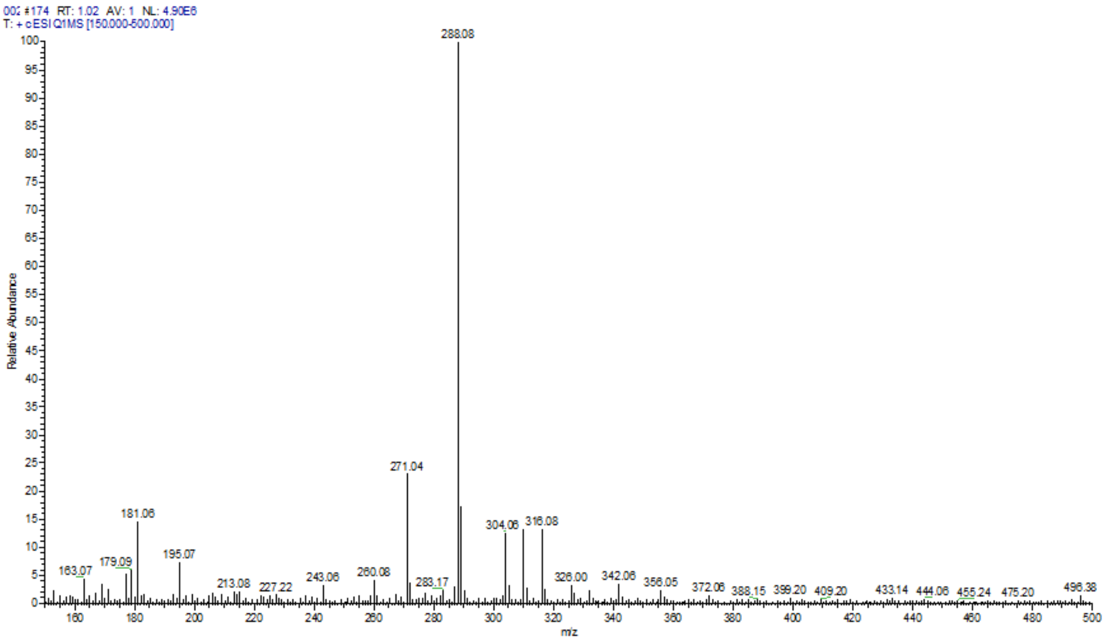

Figure S12. MS spectrum of compound 10b

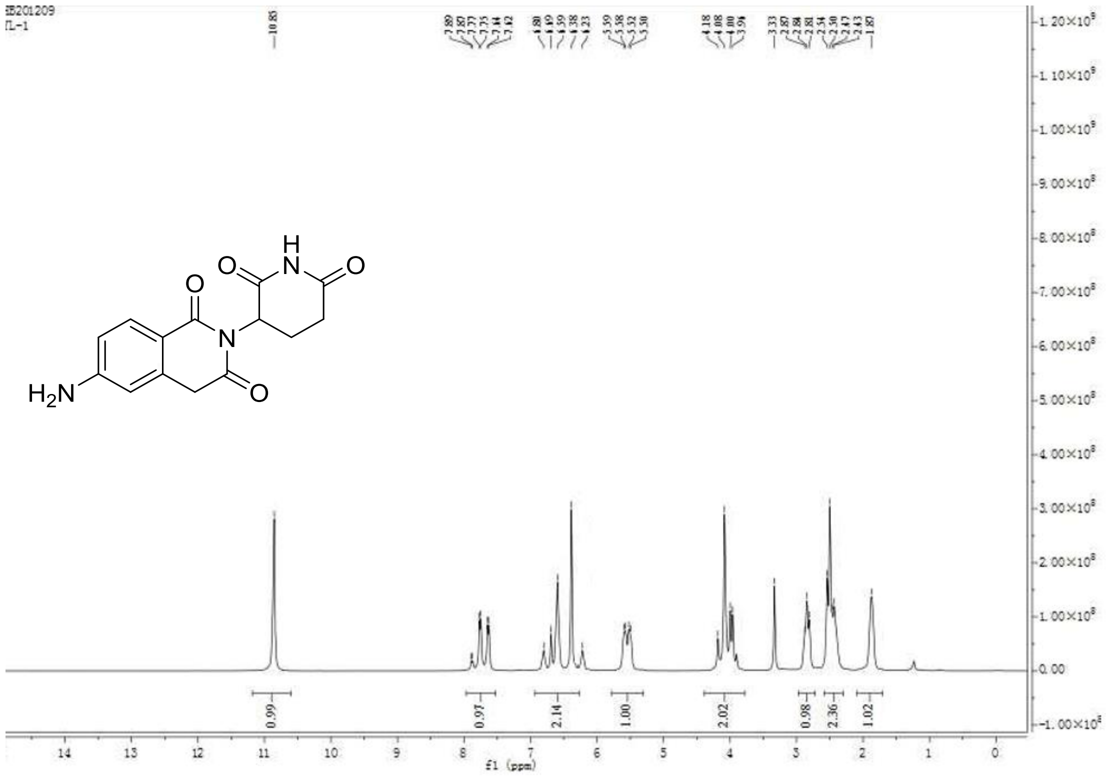

Figure S13. ^{1}H NMR spectrum of compound 10c

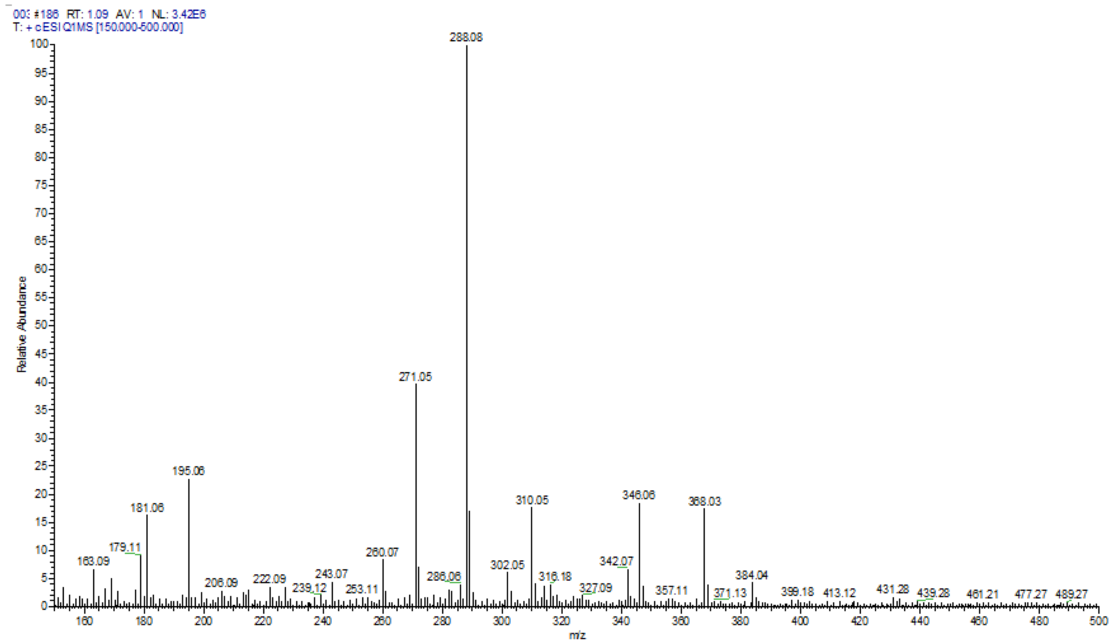

Figure S14. MS spectrum of compound 10c

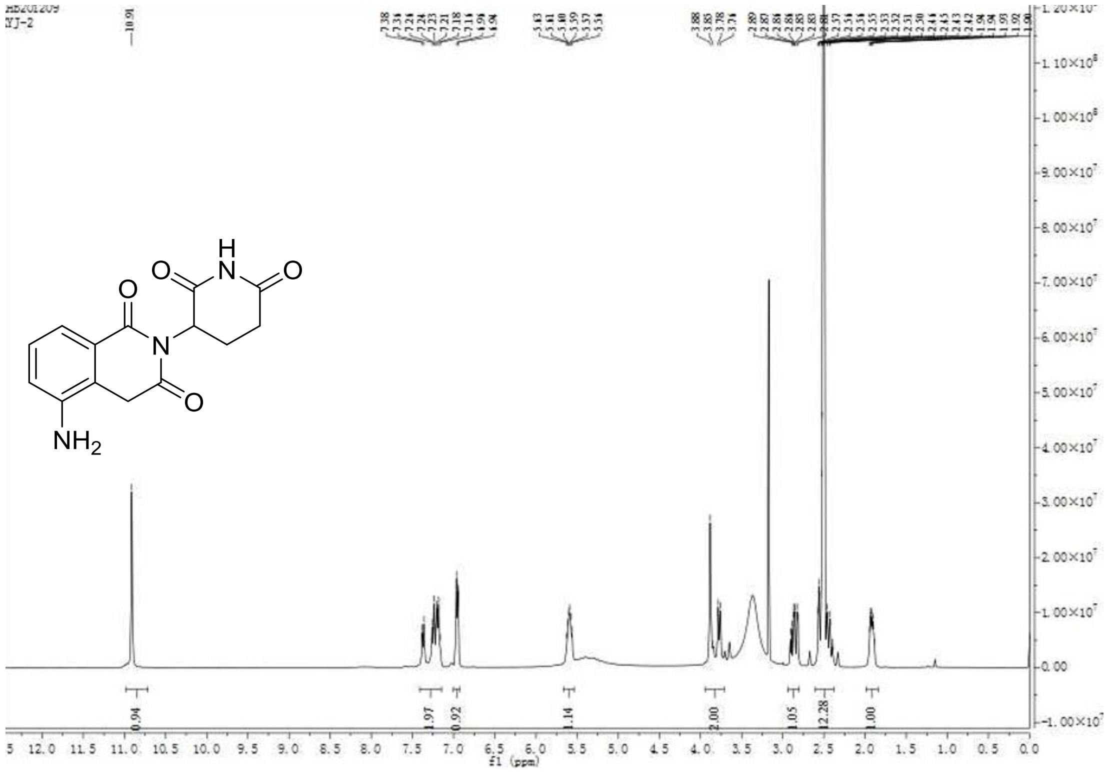

Figure S15. ^{1}H NMR spectrum of compound 10d

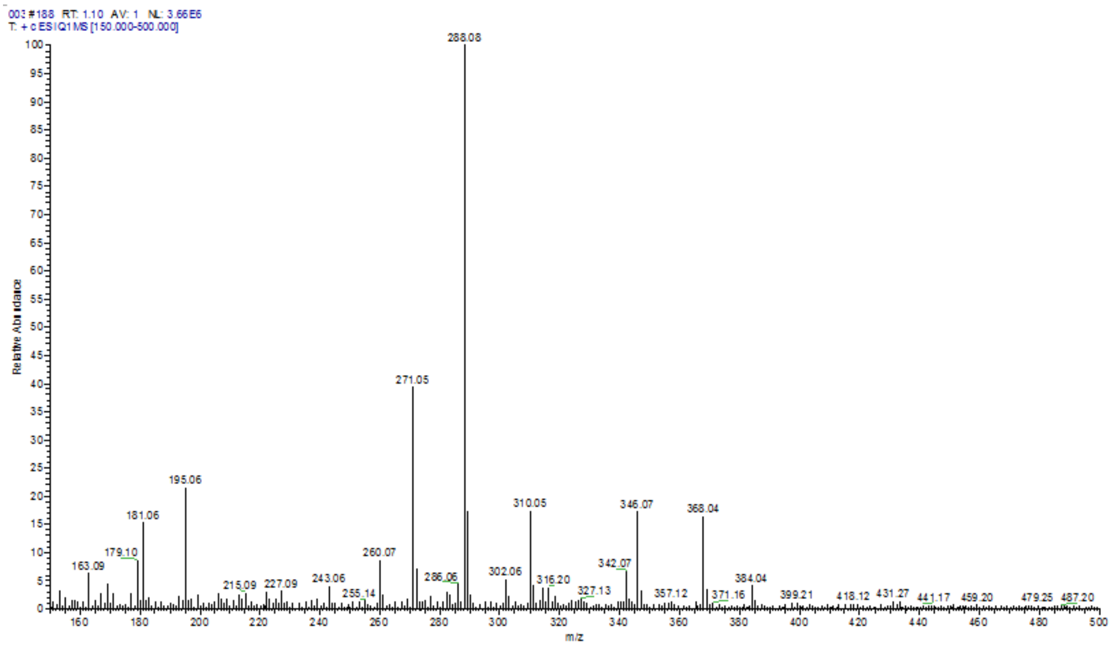

Figure S16. MS spectrum of compound 10d

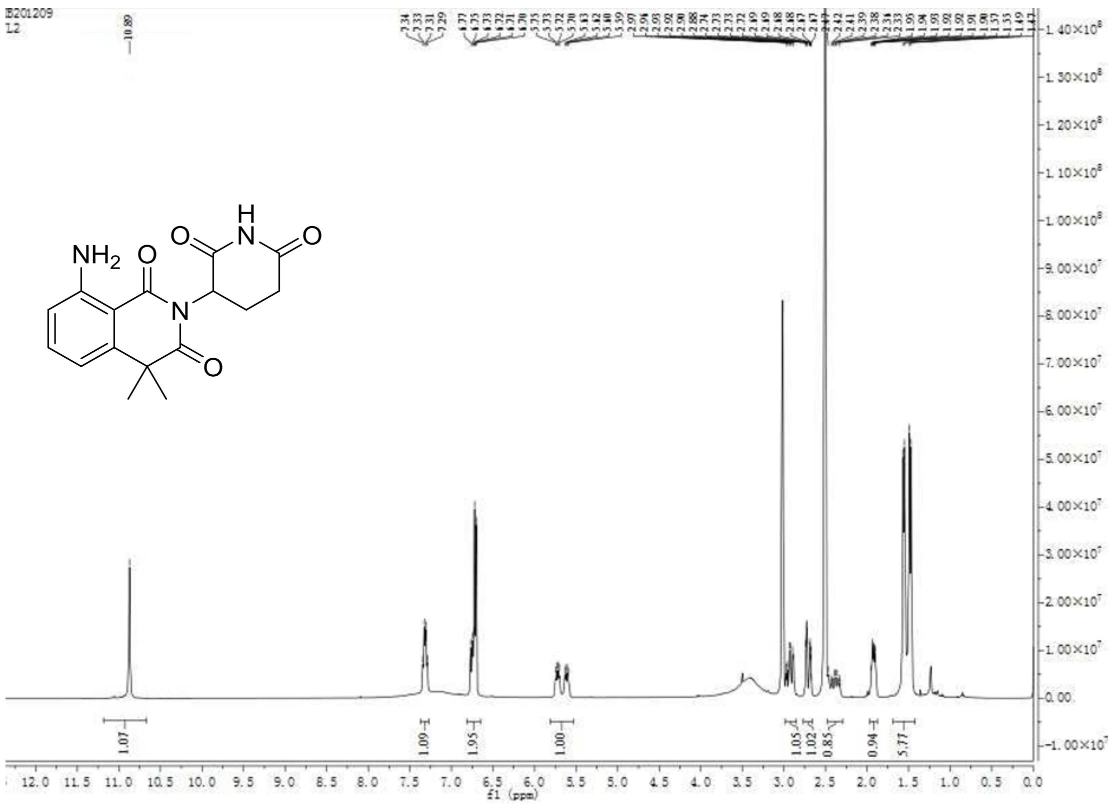

Figure S17. ^{1}H NMR spectrum of compound 12a

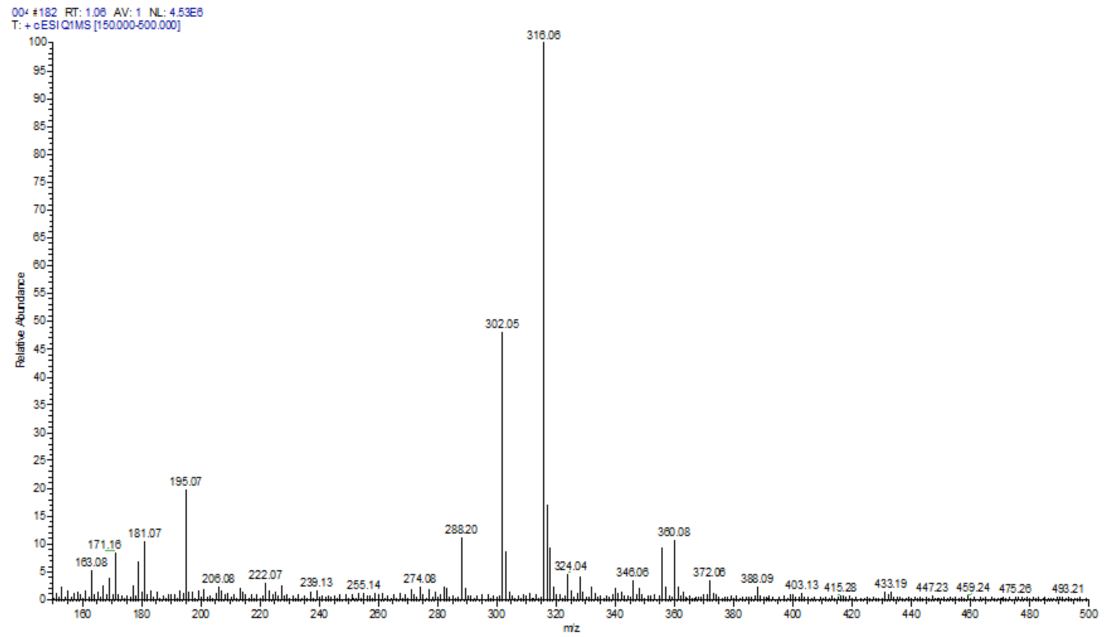

Figure S18. MS spectrum of compound 12a

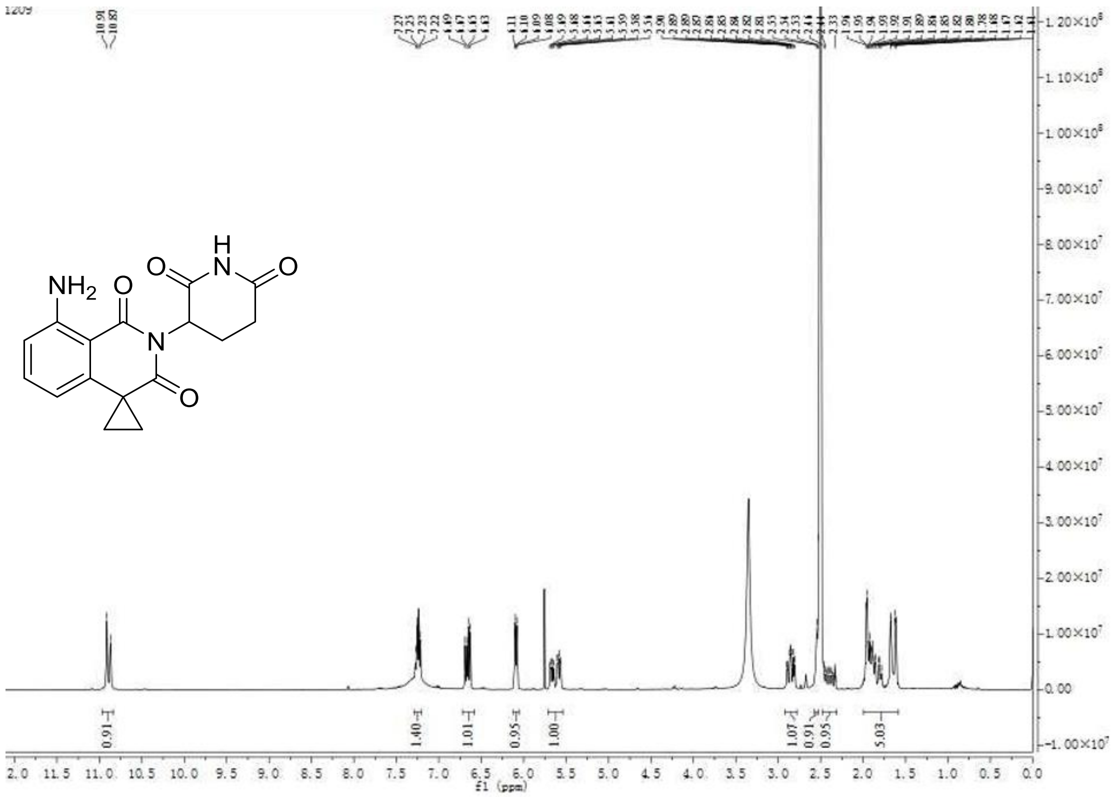

Figure S19. ^{1}H NMR spectrum of compound 12b

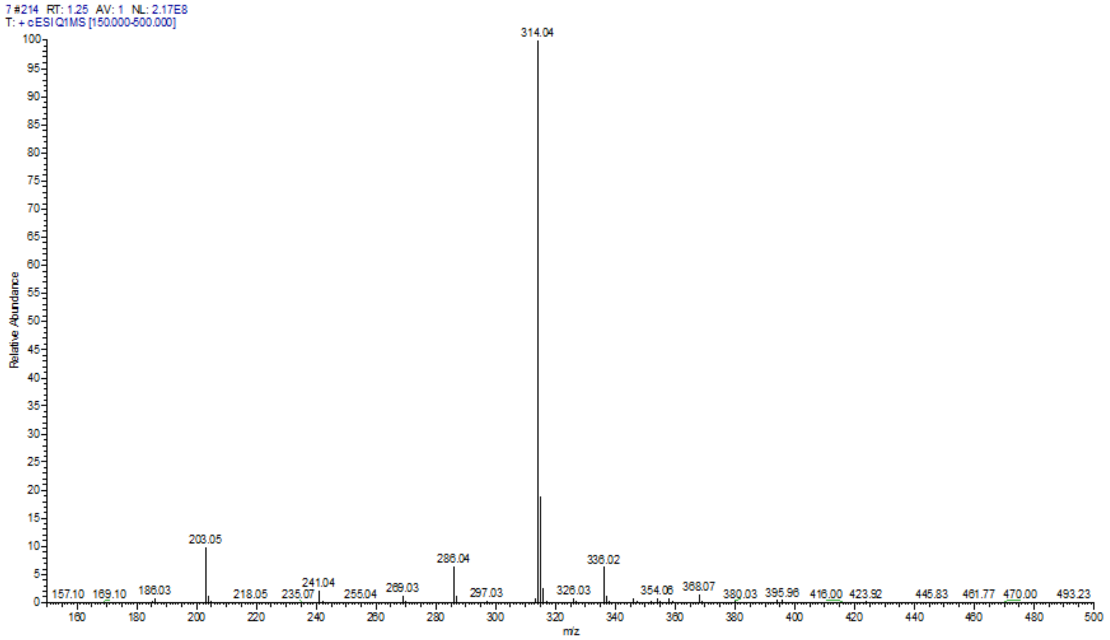

Figure S20. MS spectrum of compound 12b
